# Health-Related Quality-of-Life Profile of Pediatric Patients with β Thalassemia after Hematopoietic Stem Cell Transplantation

**DOI:** 10.3390/jcm12186047

**Published:** 2023-09-19

**Authors:** Olga Mulas, Fabio Efficace, Maria Grazia Orofino, Antonio Piroddi, Eugenia Piras, Adriana Vacca, Susanna Barella, Alessandro Costa, Johannes M. Giesinger, Giorgio La Nasa, Giovanni Caocci

**Affiliations:** 1 Hematology Unit, Businco Hospital, Department of Medical Sciences and Public Health, University of Cagliari, 09124 Cagliari, Italy; mulasolga@gmail.com (O.M.); lanasa@tiscali.it (G.L.N.); 2 Health Outcomes Research Unit, Italian Group for Adult Hematologic Diseases (GIMEMA) Data Center, 00161 Rome, Italy; f.efficace@gimema.it; 3 Bone Marrow Transplant Center, Pediatric Hospital “Microcitemico A. Cao”, 09121 Cagliari, Italy; mgorofino@tiscali.it (M.G.O.); antonio.piroddi@aob.it (A.P.); 4 Pediatric Clinic, Thalassemia and Rare Diseases, Pediatric Hospital “Microcitemico A. Cao”, 09121 Cagliari, Italy; susanna.barella@aob.it; 5 University Hospital of Psychiatry II, Medical University of Innsbruck, 6020 Innsbruck, Austria; johannes.giesinger@i-med.ac.at

**Keywords:** thalassemia, bone marrow transplantation, health-related quality of life

## Abstract

Matched hematopoietic stem cell transplantation (HSCT) is a feasible and curative treatment in pediatric patients with beta thalassemia major (β-TM). However, little data are available regarding patients and their parents’ health-related quality of life (HRQoL) after the procedure. As such, we investigated the HRQoL of pediatric patients with β-TM after HSCT compared to that of patients treated with blood transfusions and iron chelation. The health-related quality of life of 43 β-TM pediatric patients and 43 parents were evaluated using the Pediatric Quality of Life Inventory (PedsQL). A total of 25 patients underwent HSCT: 15 from a sibling and 10 from an HLA-matched donor. The median follow-up time from HSCT was 5 years (range 1–13 years). The mean ages at the survey were 10.1 years (range 5–15) and 9.6 years (range 5–15) for transfused and transplanted patients, respectively. A significant reduction in HRQoL was reported in the group of transfused patients compared with that of patients transplanted in the following PedsQL domains: children’s and parents’ physical functions, Δ = −15.4, *p =* 0.009 and Δ = −11.3, *p =* 0.002, respectively; children’s and parents’ emotional functioning, Δ = −15.2, *p =* 0.026 and Δ = −15.2, *p =* 0.045, respectively; child’s and parents’ school functioning, Δ = −25, *p =* 0.005 and Δ = −22.5, *p =* 0.011, respectively; total child and parents scores, Δ = −14.5, *p =* 0.004 and Δ = −13.2, *p =* 0.005, respectively. The results of a multivariable analysis showed that the HSCT procedure was significantly associated with a higher total child PedsQL score (adjusted mean difference = 15.3, *p =* 0.001) and a higher total parent PedsQL score (adjusted mean difference = 14.1, *p =* 0.006). We found no significant difference in the HRQoL measured after sibling or unrelated human leukocyte antigen (HLA)-matched HSCT. Finally, a significant positive correlation across all the PedsQL domains was found between the scores reported by the children and those reported by their parents. In conclusion, our study shows that HSCT in pediatric patients with β-TM is associated with a good overall HRQoL profile. This information further supports physicians when counseling patients and their parents before the HSCT procedure.

## 1. Introduction

Thalassemia is a genetic disorder that occurs when the production of α- or β-globin chains is decreased. It is a common autosomal-recessive condition, with over 50,000 new cases being reported globally each year. The prevalence of this condition is higher in regions where malaria was previously endemic, such as the Mediterranean, Africa, southeast Asia, and the Middle East [1]. Beta thalassemia major (β-TM) is a condition featuring the unequal production of the beta globin chain in hemoglobin proteins. This results in severe anemia, chronic hemolysis, extramedullary hemopoiesis, and bone deformities [2]. According to transfusion requirements, patients with thalassemia are divided into non-transfusion-dependent (NTDT) and transfusion-dependent (TDT) groups [3]. Patients with β-TM are at increased risk of developing several complications, including heart failure, splenomegaly, endocrinopathies, infectious complications, and growth impairment [4]. The unequal distribution of economic resources devoted to public health has resulted in varying life expectancies for patients with β-TM receiving treatment in developing countries.

Inadequate or insufficient levels of care are frequently encountered in these regions, unlike in industrialized countries, where β-TM patients born in recent years live nearly as long as their healthy counterparts [4,5]. Despite improvements in conventional therapies based on transfusional regimens, adequate iron chelation therapy, and multidisciplinary considerations of these patients—who are now referred to several medical specialists—disease complications and repeated access to hospitals substantially impair the health-related quality of life (HRQoL) of patients with β-TM [6]. The HRQoL is a multidimensional framework based on physical, mental, and psychosocial domains, which are evaluated based on the patient’s perception of the impact of the disease and its related treatment on their life [7]. Individuals with TD β-TM report a lower HRQoL than the general healthy population. This can be attributed to their dependence on frequent transfusions, which are associated with various complications and related treatments that affect their physical and psychological well-being. Lifelong reliance on transfusions and treatments creates a high perceived disease burden for these patients [8,9].

Hematopoietic stem cell transplantation (HSCT) remains the only curative treatment for β-TM [10,11]. HSCT in thalassemia is a procedure that replaces defective hematopoietic stem cells with healthy HSCs from a donor with identical human leukocyte antigens (HLAs). To achieve this goal, HSCs are collected from the donor’s bone marrow under general sedation and then infused in the thalassemic recipient after a conditioning regimen involving busulfan or treosulfan and cyclophosphamide. The patient undergoes immunosuppressive therapy with cyclosporine and methotrexate, generally immediately before the infusion until six months after HSCT to avoid immunologic complications such as graft-versus-host disease (GVHD).

This procedure can help many patients recover from thalassemia, especially children in good clinical condition who receive optimal transfusions and regular iron chelation treatments before a bone marrow transplant. In this category of patients, healing from thalassemia is possible in over 90% of the cases, provided a sibling or an unrelated, well-matched HLA donor is available [12]. Although HSCT should be offered as soon as possible before developing iron-related tissue damage in patients with an available HLA-identical sibling donor, the transplant procedure can be hindered by short-term complications (in particular, bone marrow rejection, infective diseases, and GVHD) and late side effects (chronic GVHD, increased incidence of solid tumors, and substantial organ dysfunctions) with impairment of the HRQoL [13]. Even if HSCT survivors achieve stable engraftment and recover from thalassemia, they may still need to take several medications, including immunosuppressants, which can increase their risk of infection and require them to frequently visit the hospital. This can make it challenging to achieve independence from the chronic condition that the bone marrow transplant was meant to overcome. A previous study reported a better 20-year HRQoL profile in a cohort of young and adult patients with β-TM who received transplants compared with that of patients treated with life-long transfusions, which was similar to that of the general population, provided their age at transplant was <15 years and they had an absence of complications such as chronic GVHD [14,15]. 

Little data are available on the HRQoL in pediatric patients with β-TM who underwent HSCT compared with that of patients with β-TM who were conventionally treated with red blood cell transfusions and iron chelation therapy [16]. Two systematic reviews and meta-analyses have recently examined the topic of HRQoL in patients with thalassemia who had undergone HSCT [17,18]. Until June 2023, only four studies published between 2008 and 2018 evaluated the HRQoL of pediatric patients who received conventional treatment or underwent transplantation. These evaluations were conducted after a median follow-up of 4.7 years since the transplant [19,20,21,22]. The HRQoL was evaluated in all four studies using the Pediatric Quality of Life Inventory (PedsQL) 4.0 Generic Core Scales, administered to patients and their parents [23,24]. Different HRQoL outcomes were identified regarding the level of correlation between children’s and parents’ reported results. Several studies in this field did not use the PedsQL for parents; in other studies, parents were found to underestimate their children’s HRQoL or reported high levels of correlation [19,20,21,22].

Notably, only a small percentage of patients, typically less than 30%, can find a suitable sibling donor within their family. In cases where a sibling donor is unavailable, a matched, unrelated HSCT is a possible alternative. Limited information is available in this context regarding the effects of unrelated donor transplants on the quality of life of pediatric patients with β-TM. Additionally, little is known about the HRQoL of pediatric patients who received transplants from siblings who were HLA-matched compared with that of patients who received transplants from unrelated HLA-matched donors.

In this study, given the lack of HRQoL data in this field, we analyzed the HRQoL collected with the PedsQL 4.0 inventory in a cohort of pediatric patients with β-TM after a median of 5 years since the bone marrow transplantation procedure. Our primary objective was to conduct a comprehensive HRQoL analysis of patients who received treatment for β-TM, comparing it with those who underwent traditional medical approaches. We analyzed the factors influencing their HRQoL, including their medical history, demographics, and whether they obtained bone marrow from a sibling or an unrelated donor. Our secondary objective was to evaluate the correlation between children’s and parents’ responses to the PedsQL 4.0 inventory. We hypothesized that patients who underwent a transplant generally have a good long-term HRQoL profile, and that no substantial differences exist between sibling and unrelated transplants in terms of HRQoL.

HRQoL remains a critical outcome for physicians and patients in counseling before undergoing a bone marrow transplantation for chronic nonmalignant diseases. Ensuring a high HRQoL after HSCT represents a pivotal outcome to be considered with children and parents in the clinical decision-making process, and further data in this field can support such a difficult choice.

## 2. Material and Methods 

### 2.1. Patients and Clinical Procedures

For this cross-sectional study, we recruited both pediatric patients who had undergone sibling or unrelated HLA-matched HSCT in the Hematology Department of Businco and Microcitemico Hospitals in Cagliari, and an age- and sex-matched control group of patients with β-TM attending the Thalassemia and Rare Diseases Clinic of the Microcitemico Hospital in Cagliari (Italy). 

The patient’s parents were informed by telephone or mail about the study’s purpose and invited to complete HRQoL and demographic questionnaires with the children through a dedicated website. 

Further clinical data (sibling or unrelated HSCT; conditioning regimen; comorbidities such as cardiovascular, metabolic, or infectious complications; acute or chronic GVHD; transfusion need; serum ferritin level; type of iron chelation; incidence of tumors) were recorded from hospital medical records.

Enrolment lasted 12 months, beginning in July 2020, and ending in July 2021. The study was approved by the Ethics Committee of Cagliari (authorization number: PG/2021/14301c). The patient’s parents provided written informed consent according to the Declaration of Helsinki.

### 2.2. Health-Related Quality of Life Evaluation 

The PedsQL 4.0 Generic Core Scale was used to assess the HRQoL [17,18]. The PedsQL 4.0 patient version was administered to patients under 18 as a self-reported questionnaire on a website and to their parents (father or mother) in the parent’s version. The instrument was administered in the version provided for the age group (5–7, 8–12, and 13–18 years).

This multidimensional questionnaire comprises 23 items and was designed to evaluate the essential core domains of pediatric HRQoL, including emotional, physical, and social functioning as defined by the World Health Organization, as well as school functioning. The PedsQL psychosocial health summary score represents the sum of items over the number of items answered in the social, emotional, and school functioning scales. The total PedsQL score is computed as the mean of the sum of all items over the number of items answered on all scales. The internal consistency, validity, and reliability of the PedsQL questionnaire have been assessed in pediatric patients with various diseases and physically healthy pediatric populations. Each item is rated on a 5-point Likert scale. The scores for each dimension of PedsQL are calculated considering the mean score, represented by the sum of the items over the number of items answered, with missing values replaced with the mean score of the remaining items. The scale score is not computed if more than 50% of the values are missing. Raw scores are transformed into standardized scores on a scale of 0–100, with higher scores representing higher levels of functioning. 

### 2.3. Statistical Analysis

Frequencies and percentages for the descriptive statistics of categorical variables and mean ± standard deviation (SD) for metric variables were calculated in both transplanted and conventionally treated groups, and compared using independent sample *t*-tests and chi-squared tests in univariate analysis. Differences in mean scores and the corresponding 95% confidence intervals (CIs) of the PedsQL scales were analyzed among transplanted thalassemia patients. 

Patients with or without HCST were compared with a linear multivariable regression model—separately for the parent’s and the child’s total PedsQL score—as dependent variables. The comparison was adjusted for clinical and demographical variables (age, sex, nationality, comorbidity, and ferritin value) if these variables remained statistically significant in stepwise variable selection.

Spearman’s coefficient was calculated to evaluate the correlation between children’s and parents’ responses. All statistical tests were two-sided with type I error α = 0.05. 

## 3. Results

### 3.1. Clinical Outcomes

Overall, 43 pediatric patients and 43 parents were enrolled in the study and completed the HRQoL survey. The clinical and demographical characteristics of patients are shown in Table 1. 

The mean age at the time of the survey was 10.1 and 9.6 years (range 5–15 years) in the group of patients with β-TM who were conventionally treated or transplanted, respectively. A total of 25 patients underwent HSCT: 15 were transplanted from a sibling HLA-matched donor, and 10 from an unrelated HLA-matched donor, resulting in complete engraftment and a transfusion-free condition. All patients underwent a myeloablative conditioning regimen based on busulphan and cyclophosphamide. The median age at transplant was 2 years (range 0–13 years); the median time interval between HSCT and the administration of the PedsQL questionnaire was 5 years (range 1–13 years). Among transplanted patients, grade II acute cutaneous GVHD was registered in three patients, and two subsequently suffered from cutaneous chronic GVHD. After transplantation, 16 (64%) patients required venesection treatment to reduce their ferritin serum level. Moreover, conventionally treated patients with β-TM continued iron chelation treatment, in most cases based on oral deferasirox. At least one comorbidity at the time of HRQoL survey compilation was recorded more frequently in conventionally treated patients (16.6% vs. 8%, *p* < 0.01). The principal comorbidities recorded were osteoporosis, diabetes, HCV infection, HBV infection, and cardiovascular comorbidities.

### 3.2. HRQoL of β-TM Transplanted Patients Compared with Those Conventionally Treated

**Figure 1:**
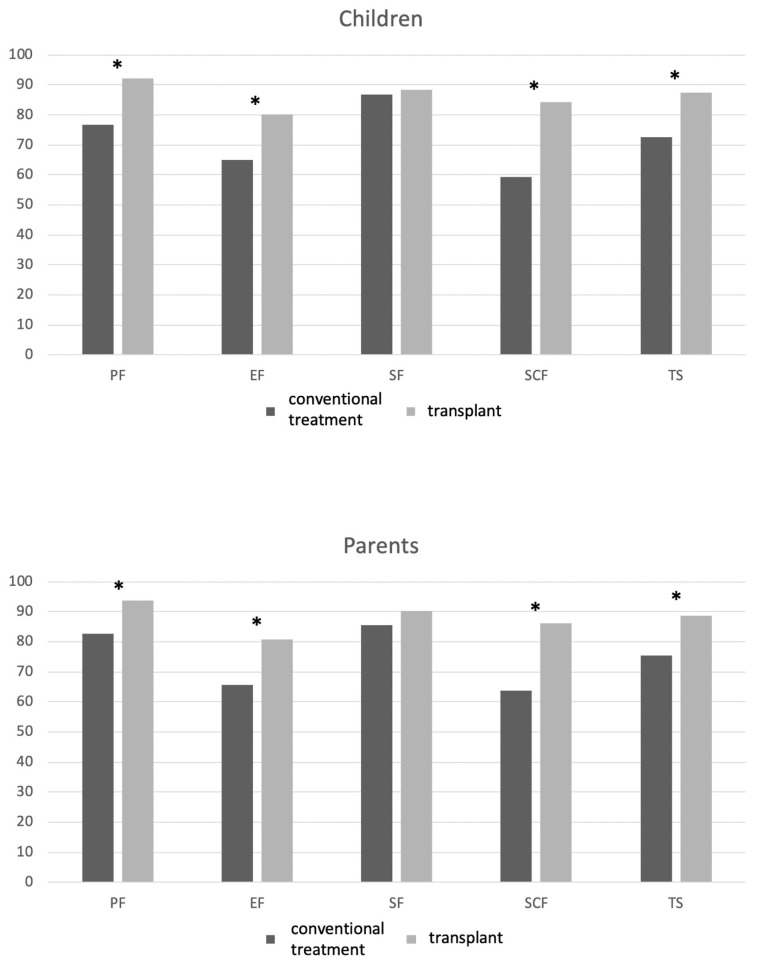
PedsQL domain scores in transplanted and nontransplanted patients with β-TM and their parents. List of abbreviations: PF, physical functioning; EF, emotional functioning; SF, social functioning; SCF, school functioning; TS: total PedsQL score. * mean difference is statistically significant (*p* < 0.05).

The differences in the mean PedsQL scores on five scales between conventionally treated and transplanted patients with β-TM and their parents’ scores are graphically displayed in Figure 1 and Table 2. 

No statistically significant differences were observed in children’s and parents’ reports for the social functioning scale. A significantly lower HRQoL was reported in all the other PedsQL domains in the group of conventionally treated patients with β-TM compared with that of the patients who underwent HSCT. In particular, significant differences in mean scores and the corresponding 95% CI were registered in the following domains: child’s and parents’ physical function, Δ = −15.4, 95% CI, −26.4 to 4.3, *p =* 0.009, and Δ = −11.3, 95% CI, −18.2 to 4.2, *p =* 0.002, respectively; child’s and parents’ emotional functioning, Δ = −15.2, 95% CI, −28.4 to 1.9, *p =* 0.026, and Δ = −15.2, 95% CI, −30.0 to −0.3, *p =* 0.05, respectively; child’s and parents’ school functioning, Δ = −25, 95% CI, −41.6 to −8.3, *p =* 0.005 and Δ = −22.5, 95% CI, −39.3 to −5.6, *p =* 0.01, respectively; total scores for children and parents, Δ = −14.5, 95% CI, −23.9 to −4.9, *p =* 0.004, and Δ = −13.2, 95% CI, −22.0 to −4.2, *p =* 0.005, respectively.

In multivariable analysis, the HSCT procedure was significantly associated with a higher total PedsQL score for children (adjusted mean difference = 15.3, SE = 4.4, *p =* 0.001) and a higher total PedsQL score for parents (adjusted mean difference = 14.1, SE = 4.8, *p =* 0.006). 

When analyzing the PedsQL domains in the group of patients who received a transplant, we found no significant differences according to the type of stem cell source (sibling vs. unrelated) (Table 3).

Finally, strong correlations of at least r = 0.81 in all PedsQL domains were found between the scores reported by children and their parents (Table 4).

## 4. Discussion 

Patients with β thalassemia experience a reduced HRQoL due to requiring lifelong blood transfusions, which require frequent hospital visits and ongoing medical treatment involving continuous chelating agents to manage blood iron overload. According to the findings, the HRQoL regarding physical and psychological well-being and distress may be comparatively lower in these patients than that of the general population [25]. Additionally, children with thalassemia who require frequent hospital visits and blood transfusions often experience a decline in their school performance. Unlike their healthy peers, this can lead to psychological distress for both children and their parents [26]. Thalassemia complications include physical deformities, delayed puberty, growth retardation, endocrinopathies, and diabetes mellitus; lower physical capacity is thus expected in most patients. However, the transfusion regimens adopted in recent years to keep pretransfusion Hb above 9–10.5 g/dL have considerably increased the life expectancy of children with β-TM. Carefully managing ferritin serum values with iron chelation therapy and implementing a collaborative approach to treatment has narrowed the gap in the life expectancy between children with β-TM and healthy individuals in developed countries.

Currently, HSCT is the only known cure for thalassemia. While gene therapy and gene editing may hold promise for future treatment options, some challenges still need to be addressed. Experimental protocols have yielded inconsistent results, and the high cost of gene transduction is a hindrance in many developing countries where thalassemia is prevalent and poses a serious social burden. Less than 100 patients underwent gene therapy worldwide compared to more than 5000 bone marrow transplants performed in several transplant centers for thalassemia, including those in the Middle East and Asia. For this reason, HSCT remains the most viable and effective curative option for patients with thalassemia at this time [1]. 

Since the 1980s, pediatric HSCT has achieved better outcomes than adult HSCT, and in 1990, the Pesaro risk score was proposed to assess transplant-related mortality in pediatric patients [27]. Patients were divided into three risk classes based on the history of inadequate iron chelation, hepatomegaly, and portal fibrosis. For patients with no risk factors, overall survival was over 90% after transplantation. Nowadays, most children with thalassemia treated in developed countries belong to the first two classes of the Pesaro risk, with optimal outcomes in the case of transplantation. Nevertheless, choosing to undergo bone marrow transplantation in a chronic nonmalignant disease remains challenging given the potential risk of mortality following the severe complications that can follow the procedure, such as infective disease, bone marrow rejection, or acute or chronic disease. When discussing the transplantation option with patients and their parents, all factors must be carefully considered. This includes evaluating the chances of recovering from thalassemia, ceasing regular transfusions, and assessing the potential risks of short- and long-term complications. 

Additionally, the impact on their daily lives must be weighed, as the transplant may enhance their sense of independence, but it may also impose a considerable burden. HSCT is currently the only treatment capable of curing β-TM and improving physical and mental health. However, information is limited on the impact of HSCT on HRQoL in the pediatric population, including for those undergoing blood transfusions, iron chelation treatments, or bone marrow transplant procedures. Table 5 summarizes the only four studies that analyzed the HRQoL collected with the PedsQL tool in pediatric patients with β-TM who received a transplant compared with that of patients who were conventionally treated until June 2023. 

Cheuk et al. evaluated a transplant β-TM group of 15 pediatric patients and a conventional treatment group of 25 patients using the PedsQL tool [20]. The median time between transplant and HRQoL assessment was 6.5 years (range 1.1–13.5 years). The researchers found no significant differences in the overall scores in the social, emotional, psychosocial, or school domains between the two groups of patients. Both groups had similar physical domain scores, but the transplanted patients reported higher running and sports exercise scores. The overall health of HSCT recipients was better than that of transfusion-dependent patients. However, whether the questionnaire was also administered to the children’s parents and the extent of the correlation between their responses remain unclear.

Caocci et al. evaluated the HRQoL longitudinal data of 28 Middle Eastern patients with β-TM using a PedsQL questionnaire before and at different time points after a bone marrow transplant (after 3, 6, and 18 months) [21]. After the transplant, the overall well-being scores from the start to 3 months after HSCT decreased. However, scores increased at 6 and 18 months after HSCT. Physical function initially worsened but then improved between 6 and 18 months after HSCT. The total PedsQL scores remained the same from baseline to 3 months after HSCT, but increased at 6 and 18 months. Social, emotional, and psychosocial scores did not significantly change following HSCT. Parents tended to underestimate their children’s HRQoL compared with their children’s self-reported scores.

Uygun et al. showed that patients who reported a higher HRQoL underwent HSCT compared with the age-matched group of patients who were nontransplanted [22]. When examining patient data by age, they found that HSCT recipients aged 2 to 4 years did not exhibit any significant differences in individual domains compared with control patients. However, in 5–7-year-old transplanted patients, a higher score was reported in the emotional domain. In the 8–12-year-old group, higher scores were observed in the physical and school domains. GVHD was identified as a significant factor, and was associated with a worse global HRQoL. The median time between the transplant and HRQoL assessment was 4.4 years (2–12 years). Based on these findings, the authors suggested that HSCT should be performed before primary school. Responses from both children and parents on the PedsQL survey were highly correlated.

Patel et al., in the most recent study, obtained similar results using the PedsQL in two Indian β-TM cohorts [19]. HRQoL was assessed at a median of 5 years (range of 2–10 years) after the transplant. The HRQoL scores of the transplanted cohort were higher than those of the control group of transfused patients, especially in the physical, emotional, psychosocial, and school domains. The type of transplant and age at which it was performed did not significantly impact their HRQoL.

Two recent systematic reviews and meta-analyses addressed the critical topic of HRQoL in patients with β-TM who underwent HSCT in pediatric and adult settings [17,18]. Only 10 studies were considered, and patients with β-TM after HSCT reported significantly higher scores in physical, emotional, and social functioning than those who were conventionally treated. Most studies were considered in the setting of hematopoietic stem cells obtained from a sibling donor [28]. In general, GVHD harms transplant patients [29]. Patients who do not experience GVHD have a higher overall quality of life, especially regarding physical and emotional functioning, pain, and insomnia [22]. In addition, after a follow-up of more than 20 years after transplantation, patients with β-TM who received a transplant reported higher scores than the general population in mental health, emotional functioning, and the cognitive domain [14]. Age at transplant was found to be another element that influences the successful outcome of the procedure. Some studies reported higher scores when HSCT was performed during pediatric age than for conventional therapy [15,22]. Mulas et al. evaluated a quantitative meta-analysis on the physical, emotional, and social function domains measured in transplanted and nontransplanted patients [17]. The result of a random-effect analysis of physical and emotional function revealed a significant difference between transplant and transfusion-dependent patients with β-TM, with a medium effect size. Effective results were found in the social functioning domain, but with a small effect size between the groups.

In our study, we collected the HRQoL after a median of 5 years after a transplant in children with β-TM who underwent HSCT compared with that of patients with β-TM treated conventionally with red blood cell transfusion and iron chelation therapy. We observed a better profile in most PedsQL domains (Figure 1, Table 2). The positive effect of HSCT on the total PedsQL score was confirmed using multivariable analysis, both in children and parents. We found a higher prevalence of comorbidities such as osteoporosis, diabetes, HCV infection, HBV infection, and cardiovascular comorbidities in the cohort of patients that were conventionally treated, which could burden HRQoL.

In particular, the most significant difference between the two groups was found in the school domain (Figure 1), with a child-reported Δ of −25 and a parent-reported Δ of −22.5. This is a notable finding because children with β-TM often complain of losing school days and having limited personal relationships because of compromised physical capacity or the burden of regular treatments and frequent hospital access. In the post-transplant period, children with β-TM who underwent a transplant need careful follow-up for at least 1 year, with periodical control as an outpatient, in particular for those presenting with complications such as chronic GVHD (in our cohort, only two patients required additional treatments for cutaneous chronic GVHD). Nevertheless, in the extended follow-up, the hospital controls of transplanted patients reduces until they return to everyday life. In a study with more than 20 years of follow-up after HSCT, we found that the cohort of ex-thalassemia patients reported daily activities and living patterns similar to those of the general population: most of the patients completed their studies after HSCT, achieving education levels in equal proportion to data stemming from the healthy population (secondary school was completed by 56% of the transplanted population and 18.3% achieved a university or higher degree) [14]. We did not find any financial burden on the HRQoL of either cohort. In Italy, medical procedures for thalassemia are entirely free of charge and are estimated to cost 40,000 EUR/year for conventional treatment and EUR 60,000 for the transplant procedure.

The study’s secondary objective was to evaluate the role of the stem cell source on the HRQoL profile of children who had received a transplant. An international consensus recommends that HSCT be offered before iron overload damage in patients with an available HLA-identical sibling donor [13]. However, less than 30% of patients can find a sibling donor within the family, depending on ethnic group and nationality. Lacking a sibling donor, a matched unrelated HSCT represents a feasible alternative, following high-resolution typing for both HLA class I and class II molecules, aiming to reduce the risk of developing immune-mediated complications such as GVHD or fatal events [10]. Our group recently published the only paper reporting data on the HRQoL in adult patients with β-TM with an unrelated transplant after a long-term follow-up (22 years) [30]. HRQoL data were collected using the FACT-SF36 tool [31]. Physical and mental domains showed a high HRQoL profile; despite older age and comorbidities in the unrelated group, no differences were found in physical or mental health domains compared with patients transplanted from a sibling donor. As expected, the acute and chronic GVHD rates were higher in unrelated transplanted patients, but this complication did not affect the long-term HRQoL. In contrast to previously published studies, in the present study, we also included HSCT from unrelated donors. We confirmed a similar long-term HRQoL profile in the pediatric setting regardless of the source of hematopoietic stem cells used for the transplant (sibling or unrelated) (Table 3). This finding is notable compared with those of previous studies in the literature. Including this aspect can help clinicians make better decisions for transplant procedures, especially when a familial donor is not a viable option.

Finally, we analyzed the level of correlation between the patient’s and parent’s reported outcomes, given contradictory or absent data reported in this field (Table 4). In a previous report where HRQoL was detected longitudinally before and after HSCT, but with a relatively short follow-up, we found that parents underestimated their children’s HRQoL. On the contrary, Uygun et al. reported a high level of correlation [22]; other studies did not mention this critical issue or did not administer the PedsQL to parents (Table 5). In our study, with an extended follow-up, we found an absolute correlation between patients’ and parents’ reported outcomes. 

Despite these encouraging results, the present study has several limitations. The number of cases considered (43 children and 43 parents) is relatively low. The study’s design was cross-sectional, and we could not compare the HRQoL profile with the baseline pretransplant values. Given the study’s exploratory nature, our results should not be interpreted as confirmatory, and further studies are needed. The long-term follow-up represents the strength of our study; moreover, we are the first to report data on the HRQoL of children with β-TM who underwent HSCT from an unrelated donor.

In conclusion, our study shows that HSCT in pediatric patients with β-TM leads to a good long-term HRQoL profile with higher satisfaction in the school domain. HRQoL represents a pivotal point that should be addressed by physicians in their counseling with patients and their parents before the HSCT procedure. 

## Figures and Tables

**Table 1:** Characteristics of patients with β-TM who received a transplant, and patients with β-TM treated with conventional therapy (blood transfusion and iron chelation).

	Conventional Therapy, N = 18	Transplant, N = 25	*p*-Value
Median age at survey (range)	10.1 (5–15)	9.6 (5–15)	0.6
Sex, male, n (%)	9 (50)	9 (36)	0.52
Type of transplant, n (%)			
Sibling		15 (60)	
Unrelated		10 (40)	
Presence of comorbidities, n (%)	3 (16.7)	2 (8)	<0.001
CV diseases, n (%)	1 (5.6)	1 (4)	0.78
Splenectomy, n (%)	0 (0)	2 (8)	<0.001
Venesection, n (%)	0 (0)	16 (64)	<0.001
RBC unit, n (%)			<0.001
none	0 (0)	25 (100)	
0–3 RBC units	10 (55.6)	0 (0)	
≥4 RBC units	8 (44.4)	0 (0)	
Ferritin level (mcg/L), mean (SD)	1293.55 (801.5)	1448.8 (527.9)	<0.001
IC, n (%)	18 (100)	3 (12)	<0.001
Oral IC	18 (100)	0 (0)	
SubC IC	1 (5.6)	3 (12)	

List of abbreviations: TM, thalassemia major; CV, cardiovascular; RBC, red blood cells; IC, iron chelation; subC, subcutaneous

**Table 2:** PedsQL domain scores in transplanted and nontransplanted patients with β-TM.

PedsQL	Conventional Therapy, Mean (SD)	Transplant, Mean (SD)	Mean Difference	95% CI	*p*-Value
Child					
Physical functioning	76.7 (21.1)	92.1 (9.6)	−15.4	−26.4; −4.3	0.009
Emotional functioning	65.0 (22.6)	80.2 (20.2)	−15.2	−28.4; −1.9	0.026
Social functioning	86.7 (14.3)	88.4 (18.9)	−1.7	−12.4; 8.9	0.745
School functioning	59.4 (29.7)	84.4 (20.6)	−25.0	−41.6; −8.3	0.005
Total score	72.6 (17.9)	87.4 (12.9)	−14.5	−23.9; −4.9	0.004
Parents					
Physical functioning	82.6 (13.5)	93.9 (8.5)	−11.3	−18.2; −4.2	0.002
Emotional functioning	65.6 (25.0)	80.8 (21.2)	−15.2	−30.0; −0.3	0.045
Social functioning	85.6 (17.5)	90.4 (17.7)	−4.8	−16.3; 6.7	0.406
School functioning	63.8 (29.1)	86.3 (17.8)	−22.5	−39.3; −5.6	0.011
Total score	75.5 (15.6)	88.6 (12.2)	−13.2	−22.0; −4.2	0.005

List of abbreviations: CI, confidential interval

**Table 3:** PedsQL domain scores in transplanted patients with β-TM according to the hematopoietic stem cell source (sibling or unrelated).

PedsQL	HSCT Sibling, Mean (SD)	HSCT Unrelated, Mean (SD)	Mean Difference	95% CI	*p*-Value
Child					
Physical functioning	93.7 (9.1)	89.6 (10.2)	+4.0	−4.0; 12.1	0.310
Emotional functioning	83.0 (17.4)	76.0 (24.2)	+7.0	−10.1; 24.1	0.408
Social functioning	93.3 (11.1)	81.0 (25.5)	+12.3	−6.5; 31.1	0.178
School functioning	91.3 (10.6)	74.0 (27.4)	+17.3	−2.7; 37.4	0.084
Total score	90.7 (8.5)	81.4 (16.5)	+9.3	−2.9; 21.7	0.124
Parents					
Physical functioning	95.2 (8.3)	91.6 (8.8)	+3.5	−3.91; 10.9	0.335
Emotional functioning	85.3 (16.8)	73.3 (26.4)	+12.0	−6.2; 30.2	0.186
Social functioning	92.3 (16.1)	87.2 (20.7)	+5.1	−10.5; 20.8	0.507
School functioning	91.0 (10.7)	78.3 (24.4)	+12.6	−2.2; 27.5	0.092
Total score	91.5 (9.5)	83.8 (15.1)	+7.7	−2.7; 18.1	0.139

List of abbreviations: CI, confidential interval

**Table 4:** Correlation of PedsQL domain scores in children and their parents.

PedsQL	Child, Mean (SD)	Parents, Mean (SD)	Correlation Coefficient	*p*-Value
Physical functioning	85.6 (17)	89.3 (12.0)	0.81	<0.01
Emotional functioning	73.8 (22.3)	74.7 (23.7)	0.95	<0.01
Social functioning	87.6 (16.9)	88.5 (17.5)	0.83	<0.01
School functioning	73.9 (27.4)	77.2 (25.2)	0.93	<0.01
Total score	80.9 (16.5)	83.3 (19.94)	0.94	<0.01

**Table 5:** Principal characteristics of studies published on HRQoL collected with the PedsQL tool in pediatric patients with β-TM after HSCT.

Author	Age of HSCT Cohort (Median, Range in Years)	Patients and Parents (No.)	Control Cohort of Conventional Treated Patients (No.)	Years of Follow Up from HSCT (Median, Range)	HRQoL Survey	Principal Findings
Cheuk et al., 2008 [20]	15.2 (5.3–18)	15/not reported	25	6.5 (1.1–13.5)	PedsQL	Emotional, social, and school domains did not differ; physical domain similar, but better scores in running and exercise in transplanted patients.
Caocci et al., 2011 [21]	10 (5–17)	28/28	longitudinal study	18 months	PedsQL	Physical function improved from 3 to 18 months after HSCT; parents underestimated their children’s HRQoL.
Uygun et al., 2012 [22]	11.6 (3.5–18)	40/40	28	4.4 (2–12)	PedsQL	Better emotional domain in 5–7-year-old and physical/school domains in 8–12-year-old transplanted patients; strong correlation between children’s and parents’ HRQoL evaluations.
Patel et al., 2017 [19]	10 (5–18)	40/40	60	5 (2–10)	PedsQL	Transplanted patients reported significantly higher scores in all domains except for social functioning.
This report	9.6 (5–15)	25/25	18	5 (1–13)	PedsQL	Transplanted patients reported significantly higher score in all domains except for social functioning; strong correlation between children’s and parents’ HRQoL evaluation.

List of abbreviations: HRQoL, health-related quality of life; HSCT, hematopoietic stem cell transplantation.

## Data Availability

Medical charts and database are available from the Pediatric Clinic, Thalassemia, and Rare Diseases, Pediatric Hospital “Microcitemico A. Cao”, Cagliari, Italy, and from the Bone Marrow Transplant Center, Pediatric Hospital “Microcitemico A. Cao”, Cagliari, Italy.
